# The association of depression and anxiety with glycemic control among Mexican Americans with diabetes living near the U.S.-Mexico border

**DOI:** 10.1186/1471-2458-14-176

**Published:** 2014-02-18

**Authors:** Darla E Kendzor, Minxing Chen, Belinda M Reininger, Michael S Businelle, Diana W Stewart, Susan P Fisher-Hoch, Anne R Rentfro, David W Wetter, Joseph B McCormick

**Affiliations:** 1 School of Public Health, The University of Texas Health Science Center, Dallas, TX, USA; 2 Population Science and Cancer Control Program, UT Southwestern Harold C. Simmons Cancer Center, Dallas, TX, USA; 3 Department of Biostatistics, The University of Texas MD Anderson Cancer Center, Houston, TX, USA; 4 School of Public Health, The University of Texas Health Science Center, Brownsville, TX, USA; 5 Department of Health Disparities Research, The University of Texas MD Anderson Cancer Center, Houston, TX, USA; 6 College of Nursing, The University of Texas at Brownsville, Brownsville, TX, USA

## Abstract

**Background:**

The prevalence of diabetes is alarmingly high among Mexican American adults residing near the U.S.-Mexico border. Depression is also common among Mexican Americans with diabetes, and may have a negative influence on diabetes management. Thus, the purpose of the current study was to evaluate the associations of depression and anxiety with the behavioral management of diabetes and glycemic control among Mexican American adults living near the border.

**Methods:**

The characteristics of Mexican Americans with diabetes living in Brownsville, TX (*N* = 492) were compared by depression/anxiety status. Linear regression models were conducted to evaluate the associations of depression and anxiety with BMI, waist circumference, physical activity, fasting glucose, and glycated hemoglobin (HbA1c).

**Results:**

Participants with clinically significant depression and/or anxiety were of greater age, predominantly female, less educated, more likely to have been diagnosed with diabetes, and more likely to be taking diabetes medications than those without depression or anxiety. In addition, anxious participants were more likely than those without anxiety to have been born in Mexico and to prefer study assessments in Spanish rather than English. Greater depression and anxiety were associated with poorer behavioral management of diabetes (i.e., greater BMI and waist circumference; engaging in less physical activity) and poorer glycemic control (i.e., higher fasting glucose, HbA1c).

**Conclusions:**

Overall, depression and anxiety appear to be linked with poorer behavioral management of diabetes and glycemic control. Findings highlight the need for comprehensive interventions along the border which target depression and anxiety in conjunction with diabetes management.

## Background

Diabetes is a leading public health concern of particular relevance to Mexican Americans. Between 2003–2006, the estimated age-adjusted prevalence of diabetes among Mexican Americans adults ≥ 20 years of age was 16.3%, compared with 9% among non-Hispanic Whites [1]. Moreover, the prevalence of diabetes was recently estimated to be over 30% among Mexican American adults residing near the U.S.-Mexico border [2]. Overweight/obesity prevalence is also alarmingly high among Mexican Americans at 81.2%, compared with 66.7% among non-Hispanic Whites [3]. Numerous studies have shown that obesity and weight gain are linked with the onset of diabetes [4-6]. Unfortunately, Mexican Americans with diabetes, particularly those born in the U.S., are at greater risk of all-cause mortality than non-Hispanic Whites [7]. In order to address diabetes-related health disparities, focused research will be needed to better understand the physiological, psychosocial, and behavioral factors that contribute to the onset and course of diabetes among Mexican Americans.

Research indicates that the relationship between depression and diabetes is bi-directional [8-13]. Depression is associated with the development of diabetes [8,12]. Conversely, diabetes is associated with the development of depression [8-10]. Given that diabetes increases risk for depression, it is not surprising that higher rates of depression are found among diabetic patients in cross-sectional studies. Further, Mexican Americans with diabetes are more likely than other racial/ethnic groups to report symptoms of depression [14]. In fact, rates of clinically significant depressive symptoms have been reported to be as high as 40% among individuals of Mexican origin with diabetes who live on either side of the South Texas border [15]. The presence of depression in diabetes is noteworthy because depression may influence glycemic control among individuals with diabetes through physiological and behavioral pathways (for reviews, see [16-19]).

Less is known about how anxiety might influence glycemic control. Initial research suggests that adults with diabetes may have elevated levels of anxiety and greater anxiety disorder prevalence relative to the general population [20-23]. Higher levels of anxiety have also been reported among Mexicans with diabetes [24]. However, initial findings related to the impact of anxiety on glycemic control among individuals with diabetes are equivocal [20,25,26]. In a meta-analysis, Anderson et al. [25] reported that the relationship between anxiety and glycemic control only approached significance, though anxiety measured via diagnostic interviews was significantly associated with hyperglycemia. Since then, Gois et al. [26] reported that anxiety (measured via self-report questionnaire) was not associated with glycemic control (i.e., HbA1c < 8 or ≥ 8). Conversely, Collins et al. [20] reported that having a high level of anxiety (measured via self-report questionnaire) was associated with poor perceived glycemic control and a greater number of diabetes complications. As such, research is needed to clarify the relations between anxiety and glycemic control in general and specifically among Latinos/Hispanics with diabetes.

Depression is associated with hyperglycemia and elevated HbA1c levels among individuals with diabetes [27-31]. Behavioral factors that contribute to poor glycemic control among individuals with diabetes may include poor adherence to recommended health behaviors including diet, weight control, physical activity, glucose monitoring, and medication regimens [17,29,32-34]. McKellar et al. [35] reported that depression had a negative impact on glucose regulation through self-care behaviors including poor adherence to diabetes dietary guidelines and poor medication adherence. Similarly, Chiu et al. [31] reported that depressive symptoms negatively influenced glycated hemoglobin (HbA1c) through diabetes-related behavioral factors including inadequate physical activity and greater body mass index (BMI). Recently, the link between depression and elevated HbA1c has been demonstrated specifically among Latinos/Hispanics with diabetes [36]. Plausibly, the same behavioral factors may be influenced by anxiety among individuals with diabetes. Additional research is needed to characterize the relations of depression and anxiety with the behavioral management of diabetes and glycemic control among Latinos/Hispanics.

The primary purpose of the current study was to evaluate the cross-sectional associations of depression and anxiety with modifiable factors known to influence glycemic control (i.e., BMI, waist circumference, and physical activity) and more direct measures of glycemic control (i.e., HbAlc and fasting glucose) among Mexican Americans with diabetes residing near the Texas-Mexico border. Potential moderators of the associations of depression and anxiety with diabetes-related factors were also evaluated including age, gender, education, preferred language, and birth country.

### Subjects

Participants were recruited from randomly selected households and invited to participate in the Cameron County Hispanic Cohort (CCHC; see [37]). Participants included in the current analyses were 492 adults ≥ 18 years of age living in the Brownsville, Texas metropolitan area on the Texas-Mexico border. The overarching CCHC design and methodology have been described elsewhere [37]. A subset of all participants with diabetes were selected for the current analyses who were born either in Mexico or the U.S. Consistent with the American Diabetes Association definition of diabetes [38], participants were included in the current study if they met one or more of the following criteria: 1) told by a doctor that he/she has diabetes, 2) currently taking a medication to manage diabetes, 3) fasting glucose ≥ 126 mg/dl, and/or 4) HbA1c ≥ 6.5. The study protocol was approved by the Committee for the Protection of Human Subjects at the University of Texas Health Science Center (HSC-SPH-03-007-B), and informed consent was obtained from all participants.

## Methods

### Measures

#### Socioeconomic status/demographic characteristics

Socioeconomic and demographic characteristics assessed included age (in years), gender, years of education, birth country (Mexico or U.S.), and preferred language of study assessments (Spanish or English).

#### Depression/anxiety

The Center for Epidemiological Studies Depression (CES-D) questionnaire is a 20-item self-report measure of depressive symptoms over the past week [39]. Items are rated on a 4-point scale, and total scores may range from 0 to 60. Scores ≥ 16 indicate clinically significant distress (will be referred to as depression throughout the manuscript). The Zung Self-Rating Anxiety Scale (SAS) is a 20-item self-report measure of anxiety [40]. Items are rated on a 4-point scale, and total scores may range from 20–80 with scores from 20–44 considered to be in the normal range. Higher scores are suggestive of greater anxiety.

#### Anthropometric characteristics

BMI was calculated based on weight and height measurements (kg/m^2^). Weight was measured without shoes to the nearest 0.2 kg using a portable electronic scale, and height was measured to the nearest 0.2 cm using a stadiometer. Waist circumference was measured with the participant in a standing position, at the level of the umbilicus to the nearest 0.2 cm.

#### Physical activity

Intensity and duration of physical activity during the last 7 days was assessed using validated instrumentation of either the International Physical Activity Questionnaire (IPAQ) [41,42] or the Godin Leisure-Time Exercise Questionnaire [43,44]. While the physical activity questionnaire changed in response to broader study design modifications, both measures assess moderate and vigorous physical activity and allow for the calculation of metabolic equivalent values (METS) using instrument-specific scoring recommendations [45,46]. METs were used as a continuous variable in the analysis. Participants who reported > 600 MET adjusted minutes in the 7 day period were considered to have met national physical activity guidelines [47]. The physical activity assessment of some participants (*n* = 92) was not concurrent with the other measures (i.e., was not completed on the same date), and instead occurred during a later participant visit. However, additional analyses controlling for the physical activity assessment (IPAQ vs. Godin vs. delayed assessment) did not substantially change the results of the physical activity models.

#### Glycemic control

Fasting glucose (mg/dl) was measured using a Glucostat Analyzer (Model 27, YSI, Inc. Yellow Springs, Ohio) following a 10-hour overnight fast. Glycosylated hemoglobin was measured on frozen whole blood using the GLYCO-Tek® Affinity column method Helena Laboratories, Beaumont, TX; [48] or by High Performance Liquid Chromatography. The validity of affinity chromatography for the determination of glycosylated hemoglobin has been demonstrated in comparison with other methods [49].

### Statistical analyses

Cross-sectional associations of depression and anxiety with BMI, waist circumference, physical activity, fasting glucose, and HbA1c were examined among Mexican Americans with diabetes in a series of linear regression models. Scatter plots of predicted values by residuals were examined to evaluate and confirm linearity. Because of the complex survey design, models accounted for socioeconomic stratum, census tract and block, and gender (for details, see [37]). Covariates included age, gender, years of education, language of study assessment (Spanish or English), and birth country (Mexico or U.S.). Current diabetes medication use (yes or no) was included as a covariate in the models where fasting glucose or HbA1c were the outcome variables. Interactions between study covariates with depression and/or anxiety were examined to determine whether any of the variables functioned as moderators of the relations between depression/anxiety and diabetes-related outcomes (i.e., BMI, waist circumference, physical activity, fasting glucose, and HbA1c). Interaction terms were created by multiplying depression and anxiety with each of the covariates. Interactions terms were evaluated for significance by including them individually in a linear regression model along with either depression or anxiety and all covariates. When an interaction term was found to be significant, the relationship between depression or anxiety with diabetes-related outcomes were further examined within either the natural groupings of the moderating variable (i.e., gender, assessment language, birth country) or the groups created by a median split for continuous variables (i.e., age, years of education).

## Results

### Participant characteristics

Participant characteristics are presented in Table 1. In summary, over 65% of participants were female, and most had less than a high school education, were born in Mexico, and elected to respond to questionnaires in Spanish rather than English. In addition, most participants were obese and appeared to have uncontrolled diabetes based on glycemic control indicators (see also [2]). Participant characteristics are presented by depression and anxiety status in Table 2. Specifically, chi-square analyses and t-tests indicated that those who experienced significant depressive symptoms within the previous week (CES-D ≥ 16) were of significantly greater age, were more likely to be female, had less education, had higher BMI and greater waist circumference, and reported less physical activity. They were also more likely to have been previously diagnosed with diabetes and to report taking diabetes medications. Similarly, participants who exhibited greater anxiety (SAS ≥ 45) were of significantly greater age, more likely to be female, had less education, were more likely to have been born in Mexico, and to have completed study assessments in Spanish. In addition, anxious participants had greater BMI and waist circumference and were less likely to meet physical activity guidelines. They were also more likely to have been previously diagnosed with diabetes and to report taking diabetes medications.

**Table 1:** Participant characteristics

	**N**	**Mean (SD)**	**%**	**Range**
Age (years)	492	51.3 (14.6)	-	18-90
Gender (% Female)	492	-	65.2	-
Education (years)	492	9.1 (5.2)	-	0-24
Birth Country (% Mexico)	492	-	66.5	-
Assessment Language (% Spanish)	492	-	78.0	-
				
BMI	488	33.2 (7.8)	-	18.3-84.7
BMI ≥ 30 (% Obese)	488	-	65.8	-
Waist Circumference (cm)	491	108.0 (16.9)	-	68-177
Physical Activity (Metabolic Equivalents)	408	921.1 (3003.3)	-	0-28,800
Not Meeting Physical Activity Guidelines (%)	408	-	76.2	-
Told by a doctor that you have Diabetes? (% yes)	492	-	57.7	-
Taking any medications for Diabetes? (% yes)	492	-	50.8	-
Fasting Plasma Glucose (mg/dl)	492	155.0 (70.2)	-	42-465
HbA1c	384	8.2 (2.3)	-	4.6-17.7
HbA1c ≥ 6.5 (%)	384	-	85.7	-
CES-D Total Score	486	13.0 (12.3)	-	0-54
CES-D Total Score ≥ 16 (% Depressed)	486	-	29.0	-
SAS Total Score	486	40.5 (8.7)	-	21-74
SAS Total Score ≥ 45 (% Mild to Extreme Anxiety)	486	-	25.5	-

**Table 2:** Characteristics of Mexican Americans with diabetes by depression and anxiety status

	**Center for Epidemiologic Studies-Depression (CES-D)**			**Zung Self-Rated Anxiety Scale (SAS)**		
	**Non-depressed (score < 16; n = 345)**	**Depressed (score ≥ 16; n = 141)**	***p***	**Non-anxious (score < 45; n = 362)**	**Anxious (Score ≥ 45; n = 124)**	***p***
Age (years)	49.79 (14.66)	55.10 (13.97)	<.001	50.15 (14.69)	54.97 (13.80)	.001
Gender (% female)	59.7	77.3	<.001	61.3	76.6	.002
Education (years)	9.62 (5.13)	7.96 (5.24)	.001	9.54 (5.27)	7.90 (4.88)	.002
Birth Country (% Mexico)	64.6	70.2	.238	63.8	73.4	.052
Assessment Language (% Spanish)	76.5	81.6	.224	75.1	86.3	.010
BMI	32.73 (7.58)	34.44 (8.17)	.029	32.50 (6.99)	35.45 (9.41)	<.001
BMI ≥ 30 (% obese)	63.2	72.1	.059	64.9	69.1	.396
Waist Circumference (cm)	106.86 (16.69)	111.12 (17.17)	.012	106.40 (15.65)	113.19 (19.31)	<.001
Physical Activity (Metabolic Equivalents)	1124.91 (3470.5)	361.0 (961.0)	.023	1061.6 (3145.2)	502.4 (2583.5)	.101
Not Meeting Physical Activity Guidelines (%)	74.7	81.1	.175	72.1	88.7	.001
Told by a doctor that you have Diabetes? (% yes)	52.2	71.6	<.001	52.8	72.6	<.001
Taking any medications for Diabetes? (% yes)	47.0	60.3	.008	45.9	65.3	<.001
Fasting Plasma Glucose (mg/dl)	151.86 (67.57)	162.48 (75.04)	.129	151.74 (67.23)	164.40 (76.62)	.082
HbA1c	8.2 (2.3)	8.3 (2.5)	.684	8.1 (2.3)	8.5 (2.5)	.118
HbA1c ≥ 6.5 (%)	86.5	83.2	.407	85.2	86.7	.733

Note: Means (standard deviations) are presented unless otherwise specified. The number of participants represented in each row may be lower than the numbers indicated at the top of column when there are missing data for the specific variable.

### Depression, modifiable factors, and glycemic control

After controlling for covariates (i.e., age, gender, years of education, assessment language, birth country), linear regression analyses correcting for design effects indicated that depression (as measured by the CES-D) was significantly and positively associated with BMI, *p* = .054 (model R^2^ = .03) and waist circumference, *p* = .005 (model R^2^ = .06), and negatively associated with physical activity (METs), *p* = .007 (model R^2^ = .03; additional analyses indicated that results remained significant even after controlling for physical activity measure). Depression was also significantly and positively associated with fasting glucose, *p* = .007 (model R^2^ = .13; see Table 3), after controlling for all previously mentioned covariates as well as medication status. Analyses indicated good fit for each model (all *p*’s < .05). Depression was not significantly associated with HbA1c.

**Table 3:** Associations between depression and indicators of glycemic control among Mexican Americans with diabetes (unstandardized coefficients [B] and standard errors [SE] are presented)

	**Modifiable factors**						**Glycemic control indicators**			
	**BMI (*N*** **= 482)**		**Waist circumference (cm; *N*** **= 485)**		**Physical activity (METs; *N*** **= 403)**		**Fasting glucose (mg/dl; *N*** **= 486)**		**HbA1c (*N*** **= 381)**	
	**B**	**SE**	**B**	**SE**	**B**	**SE**	**B**	**SE**	**B**	**SE**
Age	-.010	.026	.124*	.051	-17.791*	8.845	-.447**	.167	-.033***	.010
Gender (Male = 1; Female = 2)	.945	.715	-2.959	1.683	-595.856	337.471	-20.512**	6.471	-.379	.256
Education (years)	-.029	.084	-.149	.169	-2.894	26.881	.643	.591	-.009	.021
Assessment Language (1 = English; 2 = Spanish)	-.493	1.241	-2.155	2.573	-53.315	411.599	-.996	9.120	-.333	.367
Birth Country (1 = U.S.; 2 = Mexico)	-1.948*	.859	-5.292**	1.813	9.481	395.653	5.537	7.917	-.027	.283
Taking Diabetes Medication (0 = No; 1 = Yes)	-	-	-	-	-	-	45.387***	5.942	.823**	.270
CES-D Score (depression)	.058*	.030	.159**	.056	-20.543**	7.567	.646**	.237	.017	.010

*p ≤ .05, **p ≤ .01, ***p ≤ .001.

### Anxiety, modifiable factors, and glycemic control

After controlling for covariates (i.e., age, gender, education, assessment language, birth country), linear regression analyses correcting for design effects indicated that anxiety (as measured by the SAS) was positively associated with BMI, *p* = .001 (model R^2^ = .04) and waist circumference, *p* < .001 (model R^2^ = .08), and negatively associated with physical activity (METs), *p* = .049 (model R^2^ = .03; additional analyses indicated that results remained significant even after controlling for physical activity measure). Similarly, anxiety was positively associated with HbA1c, *p* = .047 (model R^2^ = .07), after for controlling for all previously mentioned covariates and medication status (see Table 4). Analyses indicated good fit for each model (all *p*’s < .05). Anxiety was not significantly associated with fasting glucose.

**Table 4:** Associations between anxiety and indicators of glycemic control among Mexican Americans with diabetes (unstandardized coefficients [B] and standard errors [SE] are presented)

	**Modifiable factors**						**Glycemic control indicators**			
	**BMI (*N*** **= 482)**		**Waist circumference (cm; *N*** **= 485)**		**Physical activity (METs; *N*** **= 403)**		**Fasting glucose (mg/dl; *N*** **= 486)**		**HbA1c (*N*** **= 381)**	
	**B**	**SE**	**B**	**SE**	**B**	**SE**	**B**	**SE**	**B**	**SE**
Age	-.015	.025	.114*	.050	-19.124*	8.352	-.447**	.169	-.033***	.010
Gender (Male = 1; Female = 2)	.838	.701	-3.157	1.68	-672.833*	345.717	-19.798**	6.419	-.376	.248
Education (years)	-.024	.082	-.144	.163	-.608	25.981	.571	.580	-.012	.021
Assessment Language (1 = English; 2 = Spanish)	-.800	1.188	-2.944	2.45	50.556	404.397	-3.521	9.077	-.402	.367
Birth Country (1 = U.S.; 2 = Mexico)	-1.990*	.855	-5.401**	1.817	25.134	393.21	5.477	7.933	-.045	.283
Taking Diabetes Medication (0 = No; 1 = Yes)	-	-	-	-	-	-	45.133***	6.059	.793**	.263
SAS Score (Anxiety)	.146***	.044	.336***	.090	-20.455*	10.308	.703	.389	.027*	.014

*p ≤ .05, **p ≤ .01, ***p ≤ .001.

### Moderation analyses

The interaction effects of age, gender, years of education, assessment language, and birth country with depression and anxiety on all diabetes related outcomes were evaluated (i.e., BMI, waist circumference, physical activity, fasting glucose, and HbA1c). Age, years of education, assessment language, and birth country were found to function as moderators as described below. Gender was not found to interact with depression or anxiety to predict modifiable factors related to diabetes management or glycemic control.

### Interactions with depression

After controlling for all relevant covariates, there was a significant interaction effect between depression and age on fasting glucose, B = -.040; *p* = .042. Specifically, for younger participants (< 52 years; median = 52 years of age) greater depression was associated with higher fasting glucose, while no association was found among participants of greater age (52+ years). Results also revealed a significant interaction between depression and education on HbA1c, B = .004; *p* = .004. Among more educated participants (8+ years; median = 8 years of education), depression was positively associated with HbA1c. No association was found between depression and HbA1c among less educated participants (< 8 years). Depression interacted significantly with language of assessment to predict HbA1c, B = -.004; *p* = .054. For those who completed the assessment in English, greater depression was associated with higher HbA1c. No association between depression and HbA1c was found among those who completed the assessment in Spanish. Similarly, results revealed a significant interaction between depression and birth country to predict HbA1c, B = -.052; *p* = .013. Among those born in the U.S., greater depression was associated with higher HbA1c. No association between depression and birth country was found among those born in Mexico. Please note that birth country (U.S. vs. Mexico) was highly correlated with preferred assessment language (English vs. Spanish; *r* = .622, *p* < .001).

### Interactions with anxiety

Results indicated a significant interaction between anxiety and age, B = 1.630; *p* = .005, such that anxiety was inversely associated with physical activity (METs) among participants of younger age (< 52 years; median = 52 years of age), while no association was found between anxiety and physical activity among participants of greater age (≥ 52 years). In addition, results revealed a significant interaction between anxiety, B = -.071; *p* = .034 and birth country in predicting HbA1c. Among those born in the U.S., greater anxiety was associated with higher HbA1c. However, no association between anxiety and birth country was found among those born in Mexico.

## Discussion

Overall, higher scores on measures of depression and anxiety among Mexican Americans with diabetes were associated with greater BMI and waist circumference, and engaging in less physical activity. In addition, greater depression was associated with higher fasting glucose, while greater anxiety was associated with higher HbA1c. Thus, the findings suggest that depression and anxiety have a negative influence on the behavioral management of diabetes and glycemic control among Mexican Americans residing near the U.S.- Mexico border.

Depressed/anxious participants differed from those who were not depressed or anxious in a variety of ways. Similar to nationally representative samples [50,51], depressed/anxious Mexican Americans with diabetes were of greater age, more likely to be female, and less educated than their non-depressed/anxious counterparts; they also had greater BMI and waist circumference, and engaged in less physical activity. In contrast with other research focused on anxiety disorder prevalence (e.g., [52]), anxious participants were more likely than non-anxious participants to have been born in Mexico than the U.S., and preferred to complete study assessments in Spanish. Perhaps this finding may be understood in the context of research indicating that acculturative stress is associated with greater anxiety and depression among Mexican Americans [53]. In addition, Breslau et al. [54] found that immigration from Mexico predicted the subsequent onset of anxiety. Plausibly, the higher levels of anxiety among Mexican-born participants may have been related to acculturative stress, though more research will be needed to understand potential links between nativity, acculturative stress, and anxiety.

Notably, age, years of education, assessment language, and birth country each functioned as moderators of the relationship between depression/anxiety with modifiable factors and glycemic control. Specifically, anxiety was inversely associated with hours of physical activity among younger participants, but not among participants ≥ 52 years of age. Depression was positively associated with fasting glucose among younger participants, while there was no relation among older participants. Among younger participants with diabetes, only 43% (vs. 71.2% among older participants) had been told by a doctor that they had diabetes and only 36.6% (vs. 63.8% among older participants) were taking diabetes medication. Plausibly, depression may have had a greater impact on fasting glucose among those who were undiagnosed and therefore not taking any medications to control diabetes. Conversely, the fasting glucose of those taking medications to control blood sugar may be less affected by depression.

Interestingly, greater depression and anxiety were associated with higher HbA1c among participants born in the U.S., those who were more educated, and those who completed the study assessments in English. Conversely, depression/anxiety was not associated with HbA1c among those born in Mexico, those who were less educated, and those who completed the study assessments in Spanish. Taken together, one interpretation of these findings might be that glycemic control was more influenced by depression among acculturated participants than those who were less acculturated. In the current sample, those born in the U.S. had greater education than those born in Mexico (11.84 vs. 7.71 years) and similarly, those who completed study assessments in English had greater education than those who completed assessments in Spanish (13.87 vs. 7.76 years on average). Perhaps more acculturated and educated participants had a better understanding of how to influence HbA1c through behavior, as well as greater access to diabetes-related health care (through greater education) than their less acculturated and less educated counterparts. Vega et al. [55] reported that acculturation was positively associated with cardiovascular disease-related health knowledge among Mexican Americans, and that acculturation was strongly associated with education. Thus, depression may have a greater negative impact on glycemic control among individuals who are knowledgeable about diabetes care and have been actively managing their diabetes through behavior. Alternatively, it is possible that depression may be associated with dietary responses that differ by acculturation or education. More research will be needed to understand the links between nativity, language preference, education, and glycemic control.

Given that diabetes is associated with depression and anxiety, it seems plausible that the assessment and treatment of depression and anxiety might improve self-management of diabetes and glycemic control. However, in a recent review it was concluded that while psychosocial interventions may be effective for the treatment of depression among individuals with diabetes, it is not clear that such interventions have a beneficial impact on diabetes-related outcomes [56]. Similarly, the findings of initial research suggest that pharmacological treatments for depression improve mood, but have little impact on glycemic control (for a review, see [34]). Additional studies are needed to evaluate the impact of more comprehensive interventions designed to target both depression and diabetes management, and to identify other factors that are related to both mood and glycemic control. For example, recent research suggests that depression is associated with increased risk for the onset of dementia in individuals with diabetes [57]. Finally, treatments designed to increase physical activity may be particularly beneficial for the dual purpose of reducing depression and improving glycemic control [58].

The current study has several strengths and limitations. A notable strength of the study was the sample comprised entirely of Mexican Americans with diabetes who were recruited from within the community of Brownsville, TX (on the U.S.-Mexico border), thereby avoiding the bias inherent in clinic-based studies. Latinos/Hispanics are a vulnerable and understudied group as it relates to diabetes, and even less attention has been paid to the health of those living along the border. A limitation of the study was the cross-sectional design. As such, we were not able to determine whether there might be a causal relation between depression, modifiable behavioral factors, and glycemic control among Mexican Americans with diabetes. Similarly, we could not determine whether depression precedes or develops subsequent to poor glycemic control. Plausibly, modifiable factors, such as BMI and waist circumference, may mediate the relations between depression and glycemic control among Mexican Americans as in other samples (e.g., [59]). However, given the cross-sectional nature of the data these mediational relationships were not evaluated. Finally, it is notable that the physical activity measure (from which METS were calculated) changed during the course of the study (i.e., IPAQ to Godin) and some participants completed the physical activity assessment after a delay (i.e., assessment not completed concurrently with other measures) which may have impacted the findings. However, please note that depression and anxiety remained significantly related to physical activity (METs) even after controlling for the specific physical activity measure (Godin vs. IPAQ vs. delayed assessment).

## Conclusions

Greater depression and anxiety were associated with poorer behavioral management of diabetes management and poorer glycemic control among Mexican Americans living near the U.S.-Mexico border. Findings highlight the need for comprehensive interventions designed to target depression and anxiety in the context of diabetes management among border residents. Understanding the psychosocial and behavioral factors associated with diabetes management among Latinos/Hispanics is a first step towards reducing and eliminating diabetes-related health disparities.

## Competing interest

The authors declare that they have no conflicts of interest.

## Authors’ contributions

DEK was the primary author on the manuscript and also contributed to data analysis. MC conducted study analyses and contributed to manuscript preparation. MSB, DWS, ARR, and DWW contributed to study conceptualization and manuscript preparation. BMR, SPF, and JBM contributed to study conceptualization, organization of the cohort, data collection, and manuscript preparation. JBM is the Principal Investigator on the parent study, and BMR and SPF are co-investigators. All authors read and approved the final manuscript.

## Pre-publication history

The pre-publication history for this paper can be accessed here:

http://www.biomedcentral.com/1471-2458/14/176/prepub
